# Safety and Toxicology of Cannabinoids

**DOI:** 10.1007/s13311-015-0380-8

**Published:** 2015-08-13

**Authors:** Jane Sachs, Erin McGlade, Deborah Yurgelun-Todd

**Affiliations:** Department of Psychiatry, University of Utah, 383 Colorow Drive, Salt Lake City, UT 84108 USA

**Keywords:** Cannabis, Cannabis safety, Cannabis policy, Cannabis efficacy, Cannabis

## Abstract

There is extensive research on the safety, toxicology, potency, and therapeutic potential of cannabis. However, uncertainty remains facilitating continued debate on medical and recreational cannabis policies at the state and federal levels. This review will include a brief description of cannabinoids and the endocannabinoid system; a summary of the acute and long-term effects of cannabis; and a discussion of the therapeutic potential of cannabis. The conclusions about safety and efficacy will then be compared with the current social and political climate to suggest future policy directions and general guidelines.

**Electronic supplementary material:**

The online version of this article (doi:10.1007/s13311-015-0380-8) contains supplementary material, which is available to authorized users.

## Introduction

There are more than 60 systematic reviews and meta-analyses discussing the safety, toxicology, potency, and therapeutic potential of exogenous cannabinoids. However, the general consensus of these reports is largely mixed and inconclusive. The uncertainty surrounding safety and efficacy of exogenous cannabinoids is not a product of the lack of research, but rather a product of the extreme variability in study methodology and quality. This review provides a summary of the current research on the safety and efficacy of exogenous cannabinoids, including a brief description of the chemical constituents of cannabis and how it interacts with the endocannabinoid system; a summary of what is known about the acute and long-term effects of cannabis; and a discussion of the therapeutic potential. Conclusions on safety and efficacy will then be compared with the current social and political climate in order to highlight the need for policy changes and general guidelines.

## Cannabinoids and the Endocannabinoid System

Marijuana, or cannabis, colloquially referred to as weed, pot, grass, herb, bud, ganja, and so on, is the most commonly used illicit drug both nationally and internationally. Roughly, 180.6 million people worldwide report lifetime cannabis use [1], and 24.6 million people in the USA report past-month use [2]. Cannabis is derived from the plant *Cannabis sativa*, *Cannabis indica*, or *Cannabis ruderalis*, which is includes 70 known cannabinoids, including 7 cannabigerols, 5 cannabichromenes, 7 cannabidiols (CBD), 9-Δ-9-tetrahydrocannabidiols (THC-9), 2-Δ-8-tetrahydrocannabidiols (THC-8), 3 cannabicyclols, 5 cannabielsoins, 7 cannabinols, 2 cannabinodiols, and 9 cannabitriols [3]. In addition to whole-plant cannabinoids, there are a variety of synthetic cannabinoids (e.g., dronabinol and nabilone, both synthetic THCs) and cannabinoid extracts (e.g., the oro-mucosal spray nabiximols that contain both THC and CBD) that are used both clinically and in research [4].

THC and CBD are the most commonly researched cannabinoids in the literature and there is variability in the location, mechanism, and consequences of their actions. THC has a high affinity for cannabinoid type 1 receptors (CB_1_R) [5], which are found in the highest densities in the neuron terminals of the basal ganglia, cerebellum, hippocampus, neocortex, and hypothalamus and limbic cortex [6–10]. These brain regions are involved in motor activity, coordination, short-term memory, executive function, and appetite and sedation, respectively, and it is possible that THC activity in CB_1_R in these regions may explain many of the acute effects of cannabis use [9], which will be addressed later. The endocannabinoid system also contains cannabinoid type 2 receptors (CB_2_R), which are found primarily in immune cells and tissues [6, 11]. There is evidence that activity of endogenous cannabinoids (e.g., anadamide) and exogenous cannabinoids (e.g., THC) at CB_2_R may have opposing effects on the immune system, with endogenous cannabinoids enhancing immune response and exogenous cannabinoids having immunosuppressant effects [12]. In contrast to THC, CBD has relatively low affinity for both CB_1_R and CB_2_R [5]. Despite the relatively low affinity of CBD at CB_1_R and CB_2_R, it demonstrates high potency as an antagonist. Furthermore, there is evidence that CBD may mitigate some of the effects of THC [13–15], potentially through indirect agonism, either by augmenting CB_1_R constitutional activity or endocannabinoid tone [5].

The already complex interactions of exogenous and endogenous cannabinoids in the cannabinergic system are further obfuscated by different methods of administration, inconsistent dosing measures, and highly variable cannabinoid content of cannabis plants. Cannabinoid content and consequent potency has shown extreme variance depending on the light, temperature, humidity, and soil type during cultivation, as well as genetic factors [16–19]. This is evidenced by changes in potency over time as more cannabis is grown in doors and as strains are engineered with different THC and CBD ratios [17, 19, 20]. Furthermore, the method of administration (e.g., oral, smoked, vaporized) and form of cannabinoid consumed (e.g., stems and buds, hashish, hash oil, extract, synthetic) can impact the bioavailability and consequently the response to use [4, 6, 18, 21–25]. This is particularly salient when comparing recreational and medical forms of cannabis. Collectively, these factors contribute to the difficulty in deciphering the relative safety and efficacy of cannabinoids both medically and recreationally.

## Safety: Acute and Long-term Effects

Despite the variability in research methodology and quality, there are some generalizable findings regarding the acute and long-term effects of exogenous cannabinoids.

### Effects on Physical Health

#### Cardiovascular

Cannabinoids have shown both acute and long-term cardiovascular effects. Acute dose-dependent effects of cannabis include tachycardia, increased cardiac labor, systemic vasodilation, and increased blood pressure [15, 26–29]. More severe effects such as increased angina, myocardial infarction, cardiac death, and cardiomyopathy have been recorded in individuals with pre-existing cardiovascular conditions [16, 20, 28, 30], and as such it is recommended that these individuals avoid cannabis use [31]. Paradoxically, the long-term cardiac effects of chronic cannabis use include bradycardia and hypotension, which may reflect tolerance and down-regulation over time [16, 32, 33].

#### Respiratory

Smoking is one of the main methods of cannabis administration. As such, the impact that smoking cannabis has on the respiratory system has been a point of serious concern for policy makers. A number of acute and chronic effects on the respiratory system are associated with cannabis use. Specifically, acute cannabis use has been shown to increase inflammation of large airways, increase airway resistance, and destroy lung tissue [15, 20, 29]. Further, there is evidence that chronic cannabis use also results in increased risk of chronic bronchitis [20, 29, 34], increased risk of emphysema [29], chronic respiratory inflammation [20, 26, 29, 35], and impaired respiratory function [27, 28].

#### Cancer

Although there is a pathophysiological process by which chronic cannabis use could confer an increased risk of cancer the epidemiological literature on the causal relationship is mixed [34–36]. Hashibe et al. [36] found that smoking cannabis was not associated with an increased risk of smoking-related cancers (e.g., lung, head, and neck), but might be associated with an increased risk of prostate cancer, cervical cancer, and glioma. Conversely, Reece [29] reported that smoking cannabis is associated with an increased risk of lung cancer. Other findings suggest that while cannabis does increase the risk of lung cancer, it is still lower than the risk of lung cancer associated with tobacco [20].

Comparisons of cannabis and tobacco smoke have produced mixed findings. Repp and Raich [20] found that cannabis smoke contains ammonia, hydrogen cyanide, nitric oxide, and aromatic amines at 3–5 times the rate of tobacco smoke. However, Maertens et al. [37] found that aside from the cannabinoids and nicotine, cannabis and tobacco smoke condensates contained mixtures that were qualitatively similar. They also found that cannabis smoke condensate and tobacco smoke condensate influence the same molecular processes but have subtle pathway differences that potentially account for differential toxicities and the mixed results with respect to lung cancer [37].

#### Immune System

Given the prevalence of CB_2_R in immune cells and tissues, exogenous cannabinoids likely produce immunological impacts both acutely and chronically [11]. However, the influence of exogenous cannabinoids on the immune system is multifaceted and while comprehension is improving, continued research is required [16]. While there is some evidence of immunosuppressive properties of cannabis [15, 26], there is evidence of anti-inflammatory and neuroprotective effects of CBD [13]. Specifically, CBD inhibits interleukin-10, while also increasing interleukin-8, which could have potentially therapeutic results in immune disorders [13]. The presence of both positive and negative impacts of cannabis on the immune system illustrate the potential biphasic impact on the immune system, with benefits at high and low levels and detriments at moderate levels [13, 15, 26]. Similarly, Suarez-Pinilla et al. [12] found that endocannabinoids enhanced immune response, while exogenous cannabinoids had immunosuppressant effects.

#### Sleep

There is mixed evidence regarding the impact of cannabinoids on sleep [38]. Sedation and somnolence are commonly described acute adverse effects of heavy cannabis use [4, 39–41]. Furthermore, cannabis has been shown to increase total sleep time in individuals with difficulty sleeping, including in cancer patients with chronic pain [42], individuals with post-traumatic stress disorder [43], and individuals with insomnia [41]. However, cannabis has been show to decrease slow wave sleep [6, 38]. This suggests that a consequence of the increased sleep time may be decreased sleep quality. There is also some evidence that sleep difficulty is a withdrawal symptom associated with cannabis use disorders [15, 44].

#### Effects on Cognition

It is clear that exogenous cannabinoids have an effect on cognition; however, there is considerable variability between the acute neuropsychological, chronic neuropsychological, and neuroimaging findings.

#### Acute Effects

Cannabis use has well evidenced acute impacts on cognition [13, 16, 20, 26, 34, 45–48]. Specifically, it has been reported to impair free recall [16, 20, 45], acquisition [16], working memory [15, 45], and procedural memory [20, 45]. Impairments are also demonstrated on measures of attention [15, 20, 48], impulsivity [15, 20], inhibition [49], sensory perception [26], and executive function [20, 26, 50–52]. On other measures of cognitive function the, evidence of deficits is less clear. Some studies report impairments in gross and simple motor tasks after acute cannabis use [13, 15, 16, 26, 34], whereas others find that evidence on impairments in psychomotor function is inconclusive [45]. Likewise, evidence for the impact of acute cannabis use on abstract reasoning and decision making is mixed, with some reports of impairment [20, 34], and other studies demonstrating no impact [15, 45]. Although there are clear acute cognitive effects of cannabis use, the majority are relatively short lived and diminish over time with abstinence [20, 26, 48, 53, 54].

#### Chronic Effects

Most studies have found limited evidence of persistent neuropsychological deficits among cannabis users [20, 45, 47, 51, 53–55], particularly for those who initiated cannabis use as adults [56]. However, the risk of long-term cognitive effects of cannabis use appears to increase with earlier age of onset [26, 45, 47, 48, 56–58], frequency of use [15, 34, 45, 47, 49, 59], and duration of use [15, 34, 45, 47, 49]. For instance, Repp and Raich [20] found that adolescents who initiate cannabis use before the age of 15 years demonstrate persistent pronounced deficits in visual attention, verbal fluency, inhibition, short-term recall, impulsivity, and executive functioning. Similarly, Meier et al. [46] found that cessation of cannabis did not fully restore cognitive deficits among adolescent-onset cannabis users, and may result in a greater loss of IQ in adolescence [46, 57]. Moreover, adolescent-onset users diagnosed with cannabis dependence prior to the age of 18 years were more likely to become persistent users and showed impairments in executive functioning and processing speed [46]. Other factors that may influence long-term neuropsychological effects and make between-study comparisons difficult include length of abstinence and the THC to CBD ratio [26, 45, 47]. This is particularly interesting because studies of acute administration suggest that CBD may be protective against the negative cognitive impacts of THC [45].

#### Neuroimaging Studies

In addition to the neuropsychological assessments, a number of studies have applied neuroimaging techniques to examine the effects of exogenous cannabinoids on brain structure, function, and connectivity. Recent morphological studies of adults and adolescents have found structural abnormalities in CB_1_R-rich areas, particularly in the medial temporal and frontal cortices and cerebellum, and most notably among chronic cannabis users [6, 28, 49, 60–64]. Specifically, structural neuroimaging findings suggest there are reductions in parahippocampal, hippocampal, and thalamic volume associated with chronic cannabis use when compared with healthy controls [47]. Studies of chronic adolescent cannabis users also showed structural differences in the hippocampus and amygdala [65]; gray matter volume reduction in the medial temporal cortex, temporal pole, parahippocampal gyrus, insula, and orbitofrontal cortex [6]; and reduced prefrontal volumes and white matter integrity when compared with controls [49, 60]. Studies of marijuana users who have also used alcohol or tobacco have also shown changes in brain morphometry. Heavy marijuana using adolescents with co-occurring alcohol use were found to have increased cortical thickness, particularly in frontal and parietal regions [66]. An investigation by Wetherill et al. [67] comparing adult cannabis users with and without co-occurring tobacco use reported that both groups showed smaller thalamic gray matter volume than nonusers; however, both cannabis groups and a cohort of tobacco smokers showed increased left putamen volumes. Reduced left cerebellum gray matter in nicotine users but not in cannabis users suggested that nicotine and cannabinoids exert differential effects on regional brain tissue volume [67]. Moreover, evidence suggests that in adolescents functional alterations may appear shortly after starting drug use [60]. Therefore, while cannabis use may result in morphological alteration in adults and adolescents, early onset, longer duration, and heavier use are associated with more significant alterations in structural integrity [49].

Alterations in brain function have also been observed during cannabis use. There is strong evidence that acute cannabis administration increases cerebellar and prefrontal blood flow [49, 62, 65]. However, resting state prefrontal blood flow is lower in chronic cannabis users when compared with controls [49, 60, 62]. This may represent the down-regulation of CB_1_R receptors during the resting state among chronic users [60]. Additionally, acute administration of cannabis may increase anterior cingulate cortex activity during cognitive tasks and increase brain metabolism in multiple regions during impulsivity tasks [49, 62, 68, 69]. The greater task-related activation among chronic cannabis users may reflect impaired efficiency and recruitment of additional regions [49, 60, 62, 70].

Furthermore, there is also evidence that adults who initiated regular cannabis use in adolescence may have impaired functional connectivity [34]. Specifically, neuroimaging data indicate reduced connectivity within prefrontal networks, which may be partially responsible for deficits in executive function among regular heavy cannabis uses who initiated use in adolescence [34]. These abnormalities may be explained by the influence of cannabis on the still-developing endocannabinoid system, particularly the disruption of normal pruning during adolescence when extensive re-organization of gray and white matter is occurring [57, 60]. However, while changes in white matter have been reported in adolescent cannabis users, the mechanisms for the change and long-term effects have not been fully characterized [65].

Finally, magnetic resonance spectroscopy (MRS) is a noninvasive measurement technique that enables the *in vivo* quantification of a range of neurometabolites, including γ-aminobutyric acid (GABA) and glutamate. The effects of chronic marijuana exposure have been examined through the application of MRS imaging. For example Chang et al. [71] reported reduced glutamate, choline, and myoinositol concentrations in the basal ganglia of chronic marijuana users. Applying MRS imaging, Hermann et al. [72] identified lower concentrations of *N*-acetyl aspartate in the dorsolateral prefrontal cortex of adult smokers. Prescot et al. applied recently completed a small pilot study using proton MRS to the anterior cingulate of marijuana smoking and non-smoking adolescents and also found reduced *N*-acetyl aspartate, as well as reduced glutamate and creatine in marijuana-using individuals [73].

### Effects on Mental Health

Disruptions of the cannabinergic system may have important implications for a number of neurobehavioral processes [74]. There is evidence of an association between cannabis use and both acute and chronic mental illness and psychiatric conditions, including depression, anxiety, psychosis, bipolar disorder, schizophrenia, and an amotivational state [29, 34]. However, given the variation in the disease process, as well as inconsistent cannabis dosing and composition, the full nature of the associations remains to be clarified.

In studies of cannabis use and bipolar disorder, it appears that cannabis use may exacerbate or trigger manic symptoms among individuals previously diagnosed with bipolar disorder. Gibbs et al. [75] found a 3-fold increase in risk for onset of manic symptoms after cannabis use [75]. However, there does not seem to be evidence that cannabis use is a risk factor for developing bipolar disorder [75]. Mixed findings are found when examining the relationship between cannabis use and depression and anxiety. There is some evidence that cannabis use and cannabis withdrawal may result in acute depressed mood [15, 29, 76]. Similarly, studies suggest that cannabis may increase acute anxiety [4, 15, 77]. The picture of chronic anxiety and depression and cannabis use is more complicated, in part because frequent cannabis users have both a higher prevalence of anxiety disorders, and individuals with anxiety have high rates of cannabis use [77, 78]. The fact that individuals who initiate cannabis use in adolescence may develop depression and anxiety that persists after cessation may also influence this relationship [20]. Furthermore, interpreting causality is complicated by the fact that a low concentration of THC may have anxiolytic effects, whereas higher concentrations produce anxiogenic effects [15, 77], and the evidence that CBD may mitigate the effects of THC in animal models of anxiety [5].

A significant portion of the literature dedicated to cannabis use and mental health focuses on the relationship between cannabis use and schizophrenia and psychosis. A number of studies suggest that acute cannabis exposure may induce temporary psychosis [4, 26]. Additionally, chronic cannabis use may trigger psychosis and schizophrenia in individuals with a predisposition or genetic susceptibility to mental illness [12, 20, 26, 57, 65, 79, 80]. This appears to occur in a dose-dependent manner, such that heavy cannabis use, longer duration, greater potency, and early onset of use may be more closely aligned with disease trajectory, often significantly advancing the first psychotic episode and development of schizophrenia [15, 20, 57, 65, 80, 81]. Additionally, lifetime cannabis use has been associated with higher schizotypy scores [82], and cannabis may exacerbate pre-existing symptoms of psychosis and schizophrenia [6]. Furthermore, individuals with psychosis may be more vulnerable to brain volume loss, which has been suggested as a result of cannabis exposure [6, 63, 83].

Although there is strong evidence that early cannabis use among at-risk individuals may increase the likelihood of developing schizophrenia or psychosis at a later time point, additional research is necessary to parse out the intricacies of the interaction between THC and CBD on the cannabinergic system. For example, several systematic reviews found that while cannabis use may increase subclinical symptoms of psychosis, the findings to date do not support an association between cannabis use and first psychosis [84]. Additionally, there is some evidence that cannabis with a high CBD and low THC content may mitigate psychosis [5, 85, 86].

#### Public Health and Safety

In addition to the physical, psychological, and cognitive effects of cannabis, there are clear concerns about public health and safety. A potentially serious public health effect of cannabis use is a high incidence of drugged driving and motor vehicle accidents [16, 26]. Moreover, driving impairment occurs in a dose-dependent fashion [26, 87], and individuals driving under the influence of cannabis are anywhere from 2 to 7 times more likely to be involved in both fatal and nonfatal motor vehicle collisions [20, 34].

Another risk associated with cannabis use is addiction and cannabis dependence. Nine to ten percent of individuals who initiate cannabis use will become addicted [6, 20, 34]. That number increases to 16–17 % among individuals who initiate use as an adolescent and 25–50 % among individuals who use cannabis daily [20]. The risk for addiction appears to wane as the individual ages, such that it is rarely addictive if use begins after the age of 25 years [65]. However, currently 6.5 % of twelfth graders in the USA report daily cannabis use [34], and there is evidence to suggest that as perception of harm decreases, prevalence of use increases [88]. This is particularly important because early exposure to cannabinoids may alter the reactivity of the dopamine reward centers in the brain, thereby increasing vulnerability to abuse of and addiction to other substances, and adding support to the gateway hypothesis [34, 89]. Furthermore, heavy cannabis use may be linked to negative consequences downstream, including lower income, greater need for socioeconomic assistance, unemployment, and lower life satisfaction [34].

There is also evidence of negative impacts on maternal and child health. Cannabis use during pregnancy is associated with poor physical outcomes, including birth defects, low birth weight, and an increased risk of childhood cancer, as well as poor neurodevelopmental outcomes, including aggressive behavior and attention problems in girls [20, 29, 35]. For example, children who were exposed to marijuana prenatally are more likely to demonstrate decreased problem-solving skills, as well as poor memory and attention [90, 91]. Similarly, babies exposed to marijuana prenatally show traits indicative of neurological development problems [92, 93].

## Therapeutic Potential

Despite the acute and chronic side effects of cannabis use, there is a growing body of evidence suggesting the therapeutic potential of cannabis. This is likely facilitated, in part, by the fact that certain cannabinoids, like CBD, have been well-studied and are well tolerated and safe in humans, even at high doses and chronically [94]. Exogenous cannabinoids, including nabiximols, CBD extract, and even smoked cannabis, have been recommended medically for cancer, anorexia, AIDS, chronic pain, spasticity, glaucoma, arthritis, migraine, and other illnesses for which cannabis provides relief [15, 34]. Additionally, the American Academy of Neurology published a position statement concluding that medical cannabis is ‘probably effective’ for some symptoms of multiple sclerosis (MS), including spasticity, central pain, spasms, and urinary dysfunction; is ‘probably ineffective’ for levodopa-induced dyskinesia of Parkinson’s disease (PD); and of ‘unknown efficacy’ in nonchorea symptoms of Huntington’s disease (HD), Tourette’s syndrome, cervical dystonia, and epilepsy [6, 95].

### Neurological Conditions

The literature on the therapeutic potential of exogenous cannabinoids in the treatment of MS has been the most promising. There is evidence that cannabinoids may have neuroprotective and anti-inflammatory effects in individuals with MS through the regulation of cytokine levels [96]. However, it should be noted that the degree of therapeutic potential varies according to preparation. There is evidence that oral cannabis extract and nabiximols, an oral mucosal spray containing a 1:1 ratio of CBD:THC, reduce spasticity in patients with MS [4, 6, 20, 95]; however, smoked marijuana is of uncertain efficacy [95]. Similarly, the American Medical Association found that a review of small, short-term randomized controlled trials demonstrated that smoked cannabis reduces neuropathic pain, improves appetite, and may relieve spasticity and pain in patients with MS [97]. There is also some evidence of reduced muscle stiffness, relief from pain, and improved sleep quality among patients with MS using oral cannabis extract [6]; however, these findings arise from subjective assessment of symptom relief and may be secondary to improvements in spasticity [6, 96]. While many studies suggest that cannabis may be a useful therapy for MS-related symptoms, it is important to note that not all studies assess adverse physical and cognitive impacts. Wade et al. [98] found that patients with MS who demonstrate symptom relief after use of nabiximols can continue use in the long term without tolerance, intoxication, serious adverse effects, or decrease in subjective symptomatic relief. Although the literature suggests only mild adverse physical effects associated with medical cannabis use for treatment of MS, recent studies of cannabis use in patients with MS have reported cognitive diminishment and impairment of cerebral compensatory mechanisms when compared directly with patients with MS who have not used cannabis [99–101]. Investigators from these studies raised concerns regarding the use of cannabis in a patient group with cognitive challenges prior to cannabis use.

Evidence for efficacy in other neurological conditions relies heavily on testimonials and anecdotes [20]. Animal models demonstrate the antiepileptic potential of cannabis [6, 102], and suggest that CBD may enhance the efficacy in preclinical models of epilepsy [5]. Nevertheless, there have been few controlled trials. One systematic review found that short-term daily cannabis use is safe in individuals with epilepsy, but there is currently insufficient evidence to form a conclusion about efficacy [34, 102]. Similarly, preclinical models Alzheimer’s disease, PD, and HD are mechanistically promising [6, 95, 103]. CB_2_R expression correlates with levels of β-amyloid-42 and plaque density [104]. Furthermore, cannabinoids may inhibit tau hyperphosphorylation and prevent β-amyloid aggregation [105, 106], suggesting therapeutic potential in models of Alzheimer’s disease. Similarly, the presence of striatal cannabinoid receptors on GABA terminals demonstrates a mechanism in which cannabinoids could improve dyskinesia by improving GABA transmission in the globus pallidus in patients with PD [107]. Finally, animal models of HD using cannabinoids as treatment demonstrate preservation of striatal neurons [108]. However, despite the success of these preclinical animal models, evidence from human studies remains scant [6].

### Psychiatric and Psychological Conditions

Research on the therapeutic benefits of exogenous cannabinoids on psychological conditions is equally sparse. Early clinical trials have demonstrated that high-dose oral CBD may have an anxiolytic effect, possibly through 5-HT_1A_ agonism [5, 41]. In patients with schizophrenia elevated anadamide levels are negatively correlated with psychotic symptomology, which suggests a protective role [86]. In spite of this, the benefits of CBD monotherapy are not consistently demonstrated in individuals with bipolar disorder or schizophrenia [41, 109, 110]. There is some evidence that cannabis may have a beneficial impact on sleep quality among individuals with post-traumatic stress disorder [6, 43]. However, more research is needed to confirm and further explore the therapeutic effects of cannabis or synthetic cannabinoids on psychological conditions.

### Other Medical Conditions

Some of the first conditions medicinal cannabis was approved for include glaucoma, chronic pain, and nausea and vomiting associated with cancer treatments and AIDS. There is good evidence that exogenous cannabinoids can decrease intraocular pressure in individuals with glaucoma [20, 21]. However, in order to have a clinically significant impact the dose and frequency of use needs to be extremely high, which may increase the likelihood of negative side effects [34]. Medicinal cannabis has also been used to treat chronic neuropathic pain, particularly when conventional methods do not work. Current research suggests that cannabinoids, including oral cannabis, THC, and nabiximols, provide effective analgesia [4, 6, 34, 111–113]. There is also some evidence that cannabinoids may be safe and moderately effective in the treatment of pain associated with fibromyalgia and rheumatoid arthritis [34, 42, 114]. Treatment with medicinal cannabis has resulted in decreased need for antiemetic in individuals undergoing chemotherapy [34, 39, 113, 115, 116]. Additionally, while medicinal cannabinoids (e.g., nabilone, dronabinol, and levonantradol) are not significantly better at treating nausea or vomiting than conventional medications, patients receiving chemotherapy often prefer them [34, 39, 116, 117].

Although there is evidence for the therapeutic potential of exogenous cannabinoids in the treatment of a number of conditions there is still serious trepidation regarding the potential negative side effects. A large systematic review of the adverse events associated with the use of medical cannabis demonstrated that short-term use of existing medical cannabinoids increases the risk of nonserious adverse events including mild-to-moderate sedation, dizziness, dry mouth, nausea, and poor attention [117]. The rates of serious adverse events (e.g., relapse of MS, vomiting, and urinary tract infections) were not different from controls [117]. Further research is needed to better understand the long-term effects of medical cannabis.

## Policy Perspective

### Policy Timeline and Research Limitations

Perceptions and policy regarding cannabis have vacillated widely over the years, reflecting the relative temporal valence of scientific evidence compared with public opinion at a given point in time. For example, California legalized medical cannabis in 1996, despite the federal ban [118]. Shortly thereafter, the Institute of Medicine issued a report acknowledging the potential therapeutic benefits of cannabis, but calling for more research. More recently, the pace and quality of research has been limited by stagnant policy. Cannabis was and still is categorized as a Schedule I drug, which means it is identified as potentially addictive without any medical benefit. As a Schedule I drug, the process for conducting research is extremely complicated. Researchers must have a Drug Enforcement Agency Schedule I license, approval from their institution, and funding. Obtaining all 3 is extremely challenging and has been a limiting factor in the advancement of current cannabis research. In lieu of the ability to conduct randomized controlled trials, many researchers must instead focus their efforts on retrospective cohort studies, case reports, and observational studies. There has been significant debate over the merits of re-classifying cannabis as a Schedule II drug, as it would greatly increase research accessibility and consequently methodological quality [119]. Ironically, the limited clinical research coupled with divisive public opinion and perception hinders the reclassification. In addition to reclassification, research regarding cannabis safety and efficacy is affected by a lack of standardization. Current clinical research findings are constrained by inconsistency in definitions of what constitutes a standard dose and how to quantify and standardize methods of administration [120]. Additionally, a great deal of the research relies on subjective, patient report, and patient-driven symptoms rating scales [95]. Ultimately, the federal policies remain stagnant because the process is circular; the clinical research methods and standardization are limited by the current policy, but the current policy is difficult to change because the lack of research standardization produces mixed findings.

### Public Perspective and Perceived Risk

Despite the stagnant federal policy, public perspective and perceived risk has demonstrated a noticeable shift. According to the 2012 National Survey on Drug Use and Health, cannabis is the most commonly used illicit drug in the USA with a national prevalence of cannabis use in the past month at 7 % [121]. Similarly, the Monitoring the Future Study has documented increased rates of use and decreased perceived risk between 2002 and 2012 among high-school students [88, 122]. This is further evidenced by the fact that 62 % of recent cannabis initiates were 18 years of age or younger when they first used [121]. The epidemiological evidence on use and perceived risk demonstrates a relatively clear trend in public opinion that is reflected in state but not federal policy. Despite a federal ban and limitations in the quality of evidence surrounding the potential risks associated with cannabis use, medical cannabis is currently legal in 23 states and the District of Columbia, and recreational cannabis is legal in 4 states [6, 123].

### Implementation Variation

Currently, there is state-by-state variation in the way medical cannabis legislation is designed and implemented. Specifically, there is inconsistency in the way in which states regulate patient use and access, caregiver rights, the role of dispensaries, and product safety and packaging requirements [124]. First and foremost, states can choose to enact medical cannabis legislation by 1 of 2 mechanisms, either through the introduction of statutory provisions or through the creation of an amendment to the state’s constitution. The majority of states with medical cannabis laws have opted to enact statutory provisions, in part because the process is easier, though also less stable. The next aspect of legislative design is determining who qualifies for medical use and how they obtain permission. Because physicians are subject to sanctions from the federal government, they cannot prescribe cannabis but rather must recommend use. The contexts for which a recommendation can be given vary. Some states require diagnosis of a medical condition in addition to a physician recommendation, whereas others simply require a physician recommendation [124]. After obtaining a physician recommendation there are 2 approaches an individual can follow to procure medical cannabis: home cultivation or from an approved dispensary. Currently, 15 out of 24 jurisdictions allow home cultivation; however, the circumstances under which home cultivation is permissible and the defined quantity allowed in circulation varies by jurisdiction (Table 1) [123]. Similarly, 19 out of 24 states allow dispensaries or compassionate care centers to engage in some combination of dispensing activities, including acquisition, possession, cultivation, manufacturing, delivery, transfer, selling, supplying, and dispensing of cannabis [123, 124]. Finally, there is variation in legal protections afforded to physicians, caregivers, and patients who may be involved in recommending, acquiring, or using medical cannabis. There are 2 types of protections: legal privilege that prevents the state from bringing criminal chargers, and affirmative defense that allows the individual to prevail against the criminal charges. Currently, legal protections for patients and caregivers differ from those for recommending physicians. Namely, the protections prevent the state from bringing charges against the physicians, whereas patients and caregivers may be tried but have affirmative defense that excuses criminal culpability [124].

**Table 1:** Medical and recreational marijuana legislation by jurisdiction [123]

Jurisdiction	State authorizes medical marijuana	States authorizes recreational marijuana	Affirmative defenses	Home cultivation	State regulates dispensaries	Product safety testing
Alaska	Yes	Yes	Yes	Yes	No	No
Arizona	Yes	No	No	Yes	Yes	Yes
California	Yes	No	Yes	Yes	Yes	No
Colorado	Yes	Yes	Yes	Yes	Yes	Yes
Connecticut	Yes	No	Yes	No	Yes	Yes
District of Columbia	Yes	Yes	Yes	No	Yes	Yes
Delaware	Yes	No	Yes	No	Yes	Yes
Hawaii	Yes	No	Yes	Yes	No	No
Illinois	Yes	No	No	No	Yes	Yes
Massachusetts	Yes	No	Yes	Yes	Yes	Yes
Maryland	Yes	No	No	No	Yes	No
Maine	Yes	No	Yes	Yes	No	Yes
Michigan	Yes	No	Yes	Yes	Yes	No
Minnesota	Yes	No	No	No	No	Yes
Montana	Yes	No	Yes	Yes	Yes	No
New Hampshire	Yes	No	Yes	No	Yes	No
New Jersey	Yes	No	No	No	Yes	Yes
New Mexico	Yes	No	Yes	Yes	Yes	Yes
Nevada	Yes	No	Yes	Yes	Yes	Yes
New York	Yes	No	No	No	Yes	Yes
Oregon	Yes	No	Yes	Yes	Yes	Yes
Rhode Island	Yes	No	Yes	Yes	Yes	No
Vermont	Yes	No	No	Yes	Yes	Yes
Washington	Yes	Yes	Yes	Yes	No	No

While policy should be supported by the scientific evidence, the ability to generate quality evidence thus far has been hindered by federal policy. This has resulted in a piecemeal state-based system without the ability to fully assess or implement safeguards [97]. As legalization of medical and recreational cannabis at the state level becomes more prevalent, jurisdictions are making an effort to implement safeguards, including safety testing, packaging requirements, labeling requirements, media advertisement restrictions, and distribution site features. However, of the 24 jurisdictions that have legal medical or recreational cannabis law, only 15 have product testing and regulation requirements, and only 8 have mandatory testing [123]. Therefore, while public perspective trends suggest continued state-based legislative change, the lack of federal regulation and infrastructure poses serious safety concerns.

## Discussion: Future Directions

The literature on medical and recreational cannabis suggests clear discordance between current federal and state policies, public opinion, and the scientific evidence. Moreover, this discordance appears to hinder the implementation of both high quality research and adequate safeguards.

The scientific evidence is often inconclusive and burdened by methodological inconsistency. The classification of cannabis as a Schedule I drug limits the type and quality of research, forcing assessments of safety and efficacy to rely on observational studies. Furthermore, definitions of standard dose vary, as do means of administration, cannabinoid content, potency, and reason for use (recreational and medical). Despite the methodological challenges, the findings to date illustrate relatively clear acute cardiovascular, respiratory, cognitive, psychological, and public health effects associated with both recreational and medical cannabis use. However, the documented persistence of these acute effects is considerably more variable. Long-term cardiovascular and respiratory consequences of cannabis use are fairly well evidenced. However, the findings regarding long-term cognitive, psychological, and immune effects are less clear. Few studies have assessed the long-term impact of cannabis on the immune system, and questions remain regarding the relative impacts of THC and CBD on immunity. Likewise, among healthy adults there is mixed evidence for long-term cognitive and psychological impacts of heavy cannabis use after discontinuation and washout [4, 20, 45–47, 55]. Some studies do report long-term deficits in learning and memory, but the findings are inconsistent [4, 15, 34, 45, 47, 55]. It appears that persistent cognitive diminishment and psychological impacts are closely related to early age of onset, increased duration, and frequency of use. Age of onset and frequency of use also have an impact substance abuse and dependency later on, with addiction rates of 16–17 % among individuals who initiate use as an adolescent and 25–50 % among individuals who use cannabis daily [20]. Collectively, these findings begin to depict vulnerable populations who may be negatively affected by the effects of by cannabis use, including adolescents, individuals with current or past substance use disorders, individuals with a personal or family history of mental illness, those that have compromised cardiovascular, respiratory, or immune systems, and those who are pregnant [31]. However, among the average adult user the health risks associated with cannabis use are likely no more dangerous than many other indulgences, including alcohol, nicotine, acetaminophen, fried foods, and downhill skiing [125–128]. This viewpoint is echoed in regard to medical cannabis as therapy. The side effects of conventional medications are weighted against the potential benefits, but this same logic is rarely applied to discussions of medical cannabis. This dilemma is further exacerbated by the fact that research on the therapeutic potential of cannabis relies on testimony and anecdote and is consequently heavily subjective.

Given these findings one option for the future direction of research on cannabis is to approach cannabis as a legitimate therapeutic agent. This would include reclassification, as well as more stringent and uniform supervision of its use and distribution in a safe, ethically, and scientifically justified manner [125–128]. Such policies would allow for improved research and consequently a better understanding of the safety and efficacy of cannabis.

In addition to rescheduling cannabis, further thought may be given to policy design. As state-based legalization becomes more common policymakers should consider how their policies affect production, price, and use. There is some evidence that legalization deflates production costs [129, 130], thereby potentially decreasing the market price. However, it has also been suggested that decreased cost may lead to increased use, particularly among adolescents [129]. As a result, policy makers ought to consider mechanisms to control cost. Taxes may be a useful tool to influence price and potentially adolescent use [130, 131]. Furthermore, revenue from those taxes can be utilized to promote prevention programs. Another mechanism to alter use includes limiting media promotion. Currently, only 6 jurisdictions have implemented policies restricting media advertising [123], so there is limited evidence on efficacy, but evidence from similar policies in alcohol and tobacco prevention is promising.

## Conclusion

In conclusion, despite the general uncertainty on the safety and efficacy of medical and recreational cannabis use there are some general themes that remain consistent. There are clear acute cardiovascular, respiratory, cognitive, psychological, and public health effects of cannabis use. Additionally, persistent cardiovascular and respiratory consequences are fairly well documented in chronic users. The evidence of other long-term impacts of cannabis are mixed, and likely influenced by age of first use, duration of use, frequency of use, potency, and co-morbid conditions. Finally, there is evidence suggesting a therapeutic impact of cannabis on reducing spasticity associated with MS, chronic neuropathic pain, and nausea and vomiting in individuals undergoing chemotherapy. However, studies of patients with MS who used cannabis raise a cautionary note regarding further cognitive diminishment, which may affect clinical outcomes. Therapeutic potential in the treatment of other diseases is unclear and requires more research. Collectively, these findings support the continued therapeutic use of cannabis when conventional treatments are ineffective. However, when recommending medical cannabis, physicians and patients would benefit from discussions of the risks, benefits, and uncertainties associated with cannabis use. Furthermore, medical cannabis should be avoided in vulnerable populations, including individuals under the age of 25 years, individuals with current or past substance use disorders, individuals with a personal or family history of mental illness, those that have compromised cardiovascular, respiratory, or immune systems, and those who are pregnant. Finally, efforts to reclassify cannabis should continue and policy makers must consider impacts on production, price, and use when crafting legislation.

## Electronic supplementary material

ESM 1 (PDF 653 kb)
